# Local Power: The Role of Tissue-Resident Immunity in Human Genital Herpes Simplex Virus Reactivation

**DOI:** 10.3390/v16071019

**Published:** 2024-06-25

**Authors:** Jia Zhu, Maurine D. Miner

**Affiliations:** 1 Department of Laboratory Medicine and Pathology, University of Washington, Seattle, WA 98109, USA; 2 Institute of Stem Cell and Regenerative Medicine, University of Washington, Seattle, WA 98109, USA; 3 Vaccine and Infectious Disease Division, Fred Hutchinson Cancer Center, Seattle, WA 98109, USA

**Keywords:** herpes simplex virus (HSV), HSV-2, genital herpes, subclinical shedding, tissue-resident memory (T_RM_) T cells, CD8+ T cells, T cell receptor (TCR) repertoire, dermal–epidermal junction (DEJ), tissue microenvironment

## Abstract

From established latency, human herpes virus type 2 (HSV-2) frequently reactivates into the genital tract, resulting in symptomatic ulcers or subclinical shedding. Tissue-resident memory (T_RM_) CD8+ T cells that accumulate and persist in the genital skin at the local site of recrudescence are the “first responders” to viral reactivation, performing immunosurveillance and containment and aborting the ability of the virus to induce clinical lesions. This review describes the unique spatiotemporal characteristics, transcriptional signatures, and noncatalytic effector functions of T_RM_ CD8+ T cells in the tissue context of human HSV-2 infection. We highlight recent insights into the intricate overlaps between intrinsic resistance, innate defense, and adaptive immunity in the tissue microenvironment and discuss how rapid virus–host dynamics at the skin and mucosal level influence clinical outcomes of genital herpes diseases.

## 1. Herpes Simplex Virus 2 Reactivation

Herpes simplex virus type 2 (HSV-2) causes a lifelong recurrent genital ulcerative disease that affects people worldwide [1]. Contact with infected genital secretions and microscopic breaches in the skin and mucosa allows the virus to enter the body and initiate viral replication in epithelial cells [2]. HSV-2 utilizes the peripheral nerve termini innervation network for retrograde axonal transport to sensory neurons and establishes latent infection for the lifetime of the hosts. HSV-2 reactivation can be triggered by various physical and emotional stimuli, leading to the anterograde axonal transportation of virions back to genital skin and mucosa near the original infection sites [2]. Cycling between periods of latency and reactivation, HSV-2 manifests as either an asymptomatic shedding episode or clinically apparent ulceration. Both forms of viral reactivation pose a risk of transmission to sexual partners or vertically from mother to infant [3].

The spatial and temporal patterns of HSV-2 reactivation have been characterized in depth in subjects from broad geographic regions globally. Highly sensitive and specific PCR detection combined with diary recording and self-swabbing has been utilized to monitor the reactivation of HSV-affected tissue at frequencies from once daily to multiple times per day [4,5,6,7]. These studies support the concept that HSV-2 genital reactivation is episodic, with significant variability in duration between viral shedding episodes and across individuals. The heterogeneity of HSV-2 shedding is both virological and immunological in the host tissue microenvironment [8,9]. Remarkable differences in episode length and virus titer, which can range from 10^2^ to 10^7^ DNA copies/mL, suggest that the kinetics of viral replication and the duration of reactivation vary enormously. Tissue biopsy studies have shown that lesion-forming episodes and subclinical shedding of HSV-2 reactivation can emanate from multiple anatomic sites within the same subjects on the same day [10]. Tens of thousands of lymphocytes can be found enriched in some skin loci, in contrast to the immunological paucity observed in adjacent tissues just a centimeter away [11,12]. These virological and immunological variations in the tissue microenvironment likely influence the individual variability in HSV-2 reactivation outcomes.

## 2. Tissue Resident Memory (T_RM_) T Cells

Memory T cells have been conventionally categorized into two subsets, central memory (T_CM_) and effector memory (T_EM_) T cells, according to their unique primary residence, recall responses, homing-marker expression, self-renewal potential, and effector functions [13,14]. Specifically, T_CM_ cells circulate between the spleen, bloodstream, and secondary lymphoid organs with high proliferation capacity, whereas T_EM_ cells migrate between the peripheral tissue, blood, and lymphoid organs and respond to antigen re-encountering in the periphery. More recently, tissue-resident memory T cells (T_RM_) have been identified and characterized as antigen-experienced T cells that are retained and persist at the sites of prior infection, including skin and mucosal barriers [12,15,16,17]. T_RM_ cells play a vital role in containing viral infection, owing to their ability to rapidly recognize and eliminate infected cells in nonlymphoid tissues. Identified in many organs and tissues in animals and humans, these cells typically do not re-enter circulation but remain in tissues performing immunosurveillance [18,19,20,21]. The tissue-resident memory T cell population provides on-site and rapid protection against chronic, recurrent, and latent viral infections.

**Figure 1:**
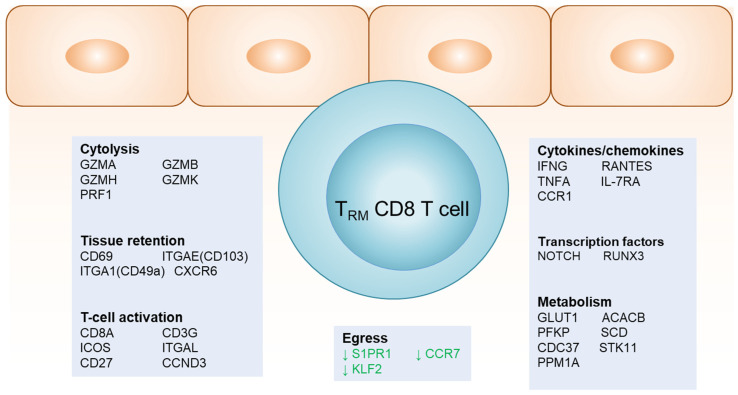
**Tissue-resident memory T cells have unique phenotypes.** CD8+ T_RM_ cells express unique gene signatures distinct from CD8+ T cells in unaffected tissue and in blood circulation. The genes listed are from human and mouse studies. Downregulated genes are in green.

T_RM_ cells are distinguished from other types of memory T cells by their signature gene expression (Figure 1). Chemokine receptors CCR7, sphingosine-1-phosphate receptor 1 (S1PR1), and their associated transcription factor KLF2, which are crucial for lymphocyte egress and recirculation, are significantly downregulated [22,23,24,25]. The upregulation of CD69 helps retain T_RM_ cells in the tissue by inhibiting S1PR1 binding to sphingosine-1 phosphate, which is abundant in the blood for promoting lymphocyte egress [26]. CD103 (encoded by *ITGAE*) and CD49a (encoded by *ITGA1*) are specific adhesion molecules important for cell retention in tissues, and their expression is upregulated [27,28,29,30]. CD103 expression facilitates T_RM_ cell retention by binding to its ligand, E-cadherin, on epithelia to limit T cells egress from the tissue [15]. As documented, not all T_RM_ cells express CD103, particularly CD4+ T_RM_ and a subset of CD8+ T_RM_ in secondary lymphoid organs and intestines [31,32,33,34]. Other core T_RM_ markers expressed by human T_RM_ CD8+ T cells include the upregulation of chemokine receptor CXCR6, the downregulation of chemokine receptor CX3CR1, and the upregulation of inhibitory molecules, CD101 and PDCD1 (PD-1) [35].

The mouse model has presented an extensive catalog of data on T_RM_ cells in response to viral infection, as longitudinal studies, experimental approaches, and tissues are readily accessible [36,37,38]. The herpes simplex virus (HSV) infection in mice has shown that T_RM_ cells collected near the epidermal site of infection are necessary for viral control [17]. This specific localization of resident T cells has also been observed in the murine model of cutaneous HSV-1 infection [28]. The use of adoptively transferred CD8+ T cells expressing the green fluorescence protein (GFP) and in vivo fluorescent imaging has shown a stochastic migration pattern of CD8+ T_RM_ localized to the site of previous infection at the epidermal interface [39]. Computational modeling based on T_RM_ velocity and diffusion calculations showed a similar retention of cells over one year. It was recently shown in mice that T_RM_ cells found at the site of HSV vaccination instigate a ‘pathogen alert’ [40], which stimulates an inflammatory response that can induce protection and decrease tissue damage (i.e., lesion severity) from a viral antigen challenge. Similarly, the vaccinia virus antigen can stimulate the T_RM_ CD8+ T-cell function when present in the localized microenvironment [41].

While the murine model of cutaneous HSV-1 infection has been extensively used to dissect the T-cell immune response to primary herpes infection [28,42,43,44], and transgenic mice have provided useful insights into primary HSV-2 infection in vaginal tissue [45,46], significant immunological differences exist between the murine model and human hosts [47,48]. Because HSV-1/HSV-2 does not undergo spontaneous reactivation in mice, this animal model is limited in understanding recurrent infection in humans. The fact that an effective HSV vaccine is still lacking, despite great promises shown in the mouse model, highlights the importance of understanding the immune responses required to restrain HSV infection in humans [49,50,51]. Here, we focus on studies of human HSV-2 infection in relation to tissue-resident immunity.

## 3. T_RM_ during Human HSV Infection

Genital herpes recurrence caused by HSV-2 infection in genital skin and mucosa provides an excellent model for studying the localized immune responses of T_RM_ cells in humans. Detailed virological and immunological studies of human genital herpes infection have led to an insightful understanding of viral spread in the skin and mucosa tissue and revealed a more dynamic interaction between the virus and human host than previously appreciated [4,6,7,52,53]. HSV reactivation in oral and genital mucosa is common, mostly subclinical, and short-lasting (<12 h), with great variability in shedding duration and virus titers between episodes and across individuals [4,5,7,54]. The dynamics of a rapidly appearing and quickly treated virus points to the importance of a targeted, localized immune response in controlling HSV-2 infection.

Several lines of evidence indicate that T-cell-mediated adaptive immunity plays a crucial role in the containment of HSV infection through vigilant monitoring and immediate response [55,56]. CD8+ T cells not only infiltrate the site of the virus encountered during ulcer-forming and productive viral infections but also persist locally after lesion resolution and tissue healing [12,57,58,59]. The persistent CD8+ T_RM_ cells are mainly distributed at the dermal–epidermal junction (DEJ), which is in close contact with basal keratinocytes and adjacent to innervating nerve endings, where reactivating viral particles are released to the epithelium. Direct cell–cell interactions are also documented between HSV-specific CD8+ T cells with the T_RM_ phenotype and HSV-latently infected neurons in human trigeminal ganglia [60,61]. Together, these spatially unique anatomic positionings imply the active interplay and communication between CD8+ T_RM_ cells and their immediate surroundings in the tissue microenvironment to attain immunosurveillance and speedy control in times of frequent viral reactivation.

HSV-2 DNA has been detected in post-healing tissue at time points of negative swabs for HSV-2, and there is no evidence of clinical disease. In this tissue of asymptomatic, subclinical HSV-2 reactivation, frequencies of HSV-specific and total CD8+ T cells are several folds higher than the tissue without viral genome detection [12]. Multiple CD8+ T cells are found in direct contact with keratinocytes expressing HSV-2 antigens. The high CD8 effector-to-target ratio and increased cytolytic granule expression provide visual proof that T_RM_ cells are capable of recognizing and aborting HSV-2 episodes and do not allow the virus to spread or undergo dissemination in the skin and mucosal barrier [59].

## 4. Gene Signatures of T_RM_ at the DEJ

DEJ CD8+ T_RM_ cells display distinctive functional pathways in clinically quiescent post-healing tissue when comparing transcription profiles to those in contralateral unaffected tissue using laser capture microdissection at the single cell level. DEJ CD8+ T_RM_ cells within normal post-healed tissue have an “activated effector T-cell” molecular signature, marked by a heightened gene expression in relation to TCR co-receptors, co-stimulatory signaling, cytolytic activity, antiviral cytokines and chemokines, G1/S cell cycle transition, and metabolic pathways [57] (Figure 1). The genetic profile notably differs from the CD8+ T cells in unaffected tissue. Activated memory immune cells, in contrast to their quiescent counterparts, require the expression of genes involved in carbohydrate and lipid metabolism that drive cell cycle progression via cellular growth and proliferation [62,63,64,65]. Specific genes associated with glucose utilization and those responsible for the expression of antiviral proteins, such as perforin and IFN-γ, contribute to an effective immune response against viral reactivation, particularly that observed in DEJ CD8+ T_RM_ cells.

*GLUT1* and *ACACB*, which are key enzymes of the glycolytic and fatty acid oxidation cycles, respectively, increase the on-site proliferation capability of activated lymphocytes as well as their induced gene expression in DEJ CD8+ T_RM_ cells, along with several other functionally related genes [57]. Thus, their upregulation substantiates the active and proliferative state of these T_RM_ cells, which is required to contain viral reactivation. The transcriptional profile of these cells also indicates a rapid turnover of effector and metabolic transcripts, such as *IFNG* and *GLUT1*, which have a half-life of <48 h, thus suggesting T_RM_ frequent encounters with viral antigens [58].

Consistent with this pathway, genes in both cytolytic and noncytolytic antiviral functional networks are specifically upregulated in DEJ CD8+ T_RM_ cells, including the antiviral effector function (*IFNG*, *GZMA*, *GZMB*); T cell receptor engagement (*CD8A*, *CD3G*); chemoattraction (*CCL5*); cell-to-cell interaction/retention (*ITGAE*); transcriptional regulation (*IRF1* and *IRF4*); and interferon-stimulated genes (*IFIT1*, *IFI16*). DEJ CD8+ T_RM_ cells also express increased levels of perforin when exposed to the HSV-2 antigen [59], suggesting that direct contact with target cells might be required for cytolytic granule production.

Moreover, DEJ CD8+ T_RM_ cells display a decreased expression of the receptors required for cell migration, such as CCR7 and S1PR1, compared to dermal CD8+ T cells near blood vessels, confirming their resident, non-migratory nature [62]. The repression of specific transcription factors, such as KLF2, associated with lymphocyte trafficking further emphasizes the non-migratory characteristics of DEJ CD8+ T cells [58]. In situ transcriptional analysis demonstrates how their residence in tissue is not constantly replenished nor capable of egressing to the blood circulation.

## 5. CD4 T Cells in HSV-2-Affected Tissue

CD4+ T cells and an array of monocytes and dendritic cells also reside in the local tissue after lesion resolution, although in a distinct spatial pattern from CD8+ T_RM_ cells. The CD4+ T cells mainly localize in the upper dermis and near blood vessels [12,33,58]. HSV-2-specific CD4 T_RM_ cells are abundant in the female reproductive tract in humans [66]. In addition, a diverse assortment of plasmacytoid dendritic cells (pDC), myeloid DC, monocytes, and macrophages localize predominantly in the dermis [58,67,68]. Cell–cell interactions between DCs and T lymphocytes were demonstrated in the genital skin and mucosa even after lesion healing [58,69] and are essential for initiating and orchestrating an effective antiviral immune response to HSV-2 infection. It has been shown that peripheral blood CD4+ T-cells and pDC responses do not correlate with herpes disease severity [70], providing further evidence that it is the localized tissue-resident immune response that affects the clinical course of human genital herpes diseases.

Interestingly, the standard treatment of the antiviral drug acyclovir for patients with a long history of genital herpes disease does not affect the CD4+ and CD8+ T cells in terms of their persistence, magnitude, distribution, and activation status, nor the local enrichment of various DCs and their interactions with resident T lymphocytes in the tissue [58]. This inflammatory response is likely due to the continuing release of the low-level HSV-2 antigen in the genital mucosa. While maintaining a local inflammatory milieu facilitates a rapid adaptive immune response to the HSV-2 antigen, it also provides fuel and targets for HIV acquisition and transmission [71,72]. The enrichment of tissue-resident CD4+ T cells and their interaction with DCs in genital HSV-2 infection provides a possible explanation for the observed increased risk of HIV infection and the ineffectiveness of acyclovir in reducing HIV acquisition in HSV-2 positive individuals [73,74,75,76].

## 6. Clonality of T_RM_ at the DEJ

The high-throughput deep sequencing of the T cell receptor (TCR) complex has made it possible to dissect, at a high resolution, the TCR repertoire dynamics in human health and disease, such as clonal composition and memory formation upon infection and vaccination [77,78]. The TCR recognizes and binds to its cognate antigen at the complementary determining region 3 (CDR3), the most hypervariable region of the molecule. Sequencing the CDR3 enables the assessment of TCR diversity, the measurement of in vivo T-cell expansion and the tracking of the frequency of individual expanded clones following a natural infection. This is particularly useful in studying the TCR dynamics in local HSV tissue infection, especially regarding the kinetics of T_RM_ formation [33,59].

TCR repertoire analysis in human genital herpes shows that T_RM_ CD8+ T cells maintain an oligoclonal nature in the skin of various anatomic locations, such as the labia, perineum, and buttocks, during and after herpes virus infection or vaccination [59,79]. Shared TCR beta chain CDR3 sequences were detected in multiple tissue biopsies over a 2.5-year period in the same individual.

There were no apparent predominant sequences between T cells from different anatomic locations [59]. The oligoclonal nature of the TCR repertoire supports the notion of chronic antigen stimulation driving clonal expansion and the long-term maintenance of antigen-specific T_RM_ CD8+ T cells in peripheral tissue during human HSV-2 reactivation. Notably, HSV-specific T_RM_ clones that predominate in the tissue are often not detectable in blood circulation, highlighting the importance of tissue-based evaluation in the development and application of therapeutic vaccinations using an HSV-specific TCR repertoire.

## 7. Impact of T_RM_ on the Epithelia Microenvironment

**Figure 2:**
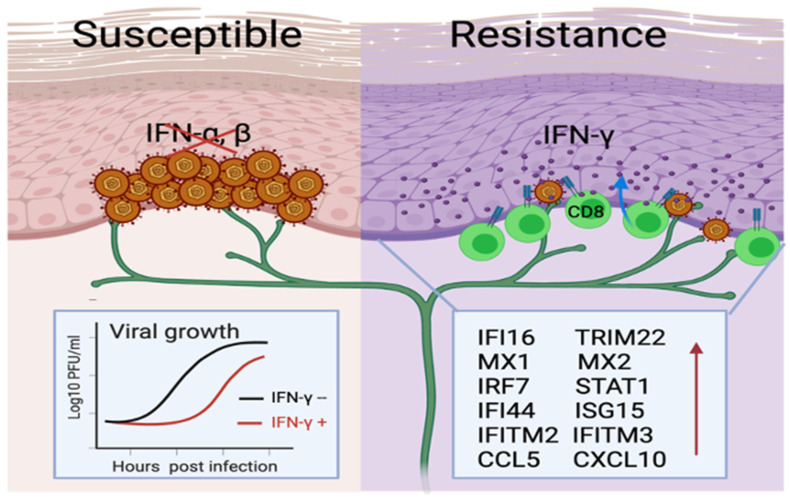
**Skin-resident memory CD8^+^ T cells influence the epidermal microenvironment.** Through IFN-γ production, DEJ CD8+ T_RM_ cells instruct the neighboring keratinocytes to induce the expression of many antiviral restriction factors to resist pathogen invasion and achieve tissue-wide protection. In the absence of CD8^+^ T cells, HSV can quickly disarm type I IFN responses and achieve productive infection, rapid growth, and ulcer formation.

The potent cytotoxic activity and high effector-to-target ratio of T_RM_ CD8+ T cells have been implicated as underlying mechanisms for rapid clearance and early containment during asymptomatic HSV-2 reactivation. Questions arise regarding how tissue-wide protection is achieved on the entire barrier surface by a handful of resident T cells and whether containment can be attained non-cytolytically. T_RM_ CD8+ T cells in mouse skin demonstrate the induction of a pathogen alert at the previously infected site that can be amplified to protect the entire skin tissue [40]. In situ studies in human genital HSV-2 infection reveal a robust immunological crosstalk between DEJ T_RM_ CD8+ T cells and their neighboring epithelial cells [80]. This study supports the notion that DEJ T_RM_ CD8+ T cells, through recognizing virally infected cells at the specific HSV-2 peptide and HLA level, instruct broad innate and cell-intrinsic antiviral defense mechanisms and activate IFN-related responses in the surrounding microenvironment (Figure 2).

IFN-γ produced by DEJ CD8+ T cells induces a core gene signature in primary human keratinocytes involving signaling pathways in antigen presentation (*B2M*, *HLA-A*, and *MICB*), chemoattraction (*CXCL10*, *CCL5*, and *CCL8*), and intracellular antiviral restriction (*IFITM1*, *IFITM2*, *IFITM3*, *MX1*, *MX2*, *ISG15*, *HERC5*, *STAT1*, *IFI16*, *TRIM22*, *BST2* and *RIG-I*) [81]. HSV-2 infection alone elicits a poorly antiviral gene expression and even antagonizes IFN-γ-stimulated genes (*MX2*, *ISG15*, *HERC5*, and *UNC93B1*), which is consistent with the viral immune evasion of type 1 IFN immunity [81]. Genetic studies indicate that humans with mutations in *STAT1* and *UNC93B* are susceptible to HSV encephalitis [82,83]. The fact that type 1 IFN genes are rarely detected in HSV-2-affected tissue during clinical ulcer-forming lesions and post-healing phases [68] further supports the critical roles of T_RM_ cells via cytokine production mediating innate and cell-intrinsic antiviral immunity to eliminate HSV-infected cells [84].

The broad spectrum of restriction factors elicited by IFN-γ effectively blocks HSV immediate-early gene transcription, which is the earliest step in the HSV life cycle [59,85]. Interference with viral gene expression can lead to resistance to pathogen invasion and result in an extended lag time before the onset of infectious virus production, translating into a hundred-to-thousand-fold reduction in the virus burden. This delay in viral growth might offer the host a significant advantage in mounting immune defenses and potentially altering clinical outcomes from an active lesion to subclinical reactivation. Thus, T_RM_ CD8+ T cells establish an antiviral “field effect” via mediating immune crosstalk to influence neighboring tissues and beyond, ultimately contributing to the successful host containment of viral infections (Figure 2).

Together, T_RM_ cells, through antigen-specific recognition, engage both the cytolytic effector function and cytokine-driven communication to orchestrate cell-intrinsic and innate immune responses within tissue microenvironments, establishing a feedback loop connecting adaptive and innate immune mechanisms for tissue protection.

## 8. Concluding Remarks

Tissue-resident memory T cells in human genital herpes infection leave a long-term, previously unappreciated subclinical inflammatory response in the periphery of normal-appearing genital skin and mucosa. The magnitude, function, and spatial distribution of such local immunity can significantly influence the frequency and clinical outcomes of HSV-2 reactivation. The localization of these sentinel T_RM_ cells at the gateway of the reactivated virus released from the sensory nerve endings, along with their activation signatures and TCR clonal pattern, all point to DEJ T_RM_ CD8+ T cells reacting to and containing the HSV-2 viral infection in the peripheral tissue before recurrent disease occurs. It remains to be elucidated whether the enormous heterogeneity of HSV-2 shedding severity documented in clinical trials is due primarily to fluctuations in T_RM_ CD8+ T cell frequency in tissues at the episode onset. What are the cellular factors, surface molecules, and metabolites expressed by the surrounding skin and mucosa influencing the activation, function, proliferation, and survival of T_RM_ CD8+ T cells? These are all important concepts in developing novel immunotherapeutic approaches to chronic viral infections.
